# Autodialysis in Morocco: how much longer can we wait?

**DOI:** 10.11604/pamj.2019.33.162.13282

**Published:** 2019-07-03

**Authors:** Béfa Noto-Kadou-Kaza, Hissein Ali Mahamat, Naoufal Mtioui, Amina Izem, Lalla Meryam Abouamrane, Selma El-Khayat, Mohamed Zamd, Ghislaine Medkouri, Mohamed Gharbi Benghanem, Benyounes Ramdani

**Affiliations:** 1 Departement of Nephrology, Dialysis and Renal Transplantation of University Teaching Hospital Ibn Rochd of Casablanca, Casablanca, Morocco

**Keywords:** Autodialysis, auto-dialysis, self-dialysis, home hemodialysis, dialysis, dialysis accession, renal replacement therapy (RRT)

## Abstract

**Introduction:**

Autodialysis is the dialysis performed by the patient himself at a local center instead of a hemodialysis center. In Morocco, the practice of hemodialysis dates back to 1970; however, an autodialysis center does not yet exist. The objective was to assess the potential medical fitness and adherence of the patients to an autodialysis program.

**Methods:**

Descriptive and analytical multicenter study conducted in March 2015 involving patients from of eight hemodialysis centers in Casablanca (Morocco). The study was conducted in two steps: 1) a transversal assessment of the medical potential to achieve autodialysis that included 556 patients; 2) a survey of the autodialysis membership that included 383 out of 556 patients who were deemed eligible for autodialysis.

**Results:**

The average age was 54.63 ± 15.16 years; the average of hemodialysis duration was 85.9 ± 78.1 months. Diabetic nephropathy (22.7%) was the predominant cause of kidney disease. The assessment of medical potential to achieve autodialysis highlighted that almost all of the patients were in good condition (93%), independent (81%), and those without major comorbidities were less than 76 years old. Regarding the potential patients' adherence to autodialysis, among the 383 patients previously deemed suited for autodialysis, 293 (76.5%) responded favorably to the proposal of self-dialysis.

**Conclusion:**

The practice of hemodialysis should be implemented in a short time in Morocco because our patients' profile is perfectly suitable to this therapeutic method especially when they are young, in good general condition, autonomous, without major comorbidities, and willing to learn.

## Introduction

The term "autodialysis" refers to the dialysis performed by the patient himself in a local center instead of a hemodialysis hospital center where the dialysis session requires the assistance of medical staff. Therefore, autodialysis is a technique of self-treatment for patients with kidney disease. Unlike hemodialysis centers, self-dialysis centers are located in small units where there is one nurse per 6-8 patients, and a physician, who is available on 24 hours per day, visits the patients at least one time per month during dialysis sessions [1]. These self-dialysis centers are organized for patients who have a certain degree of autonomy, are young, and without significant comorbidities. In the world, autodialysis has been practicing for decades; for example, in France, it has been performing for more than 30 years and in 2012 its prevalence was 25% [2]. The resort of this technique continues to increase around the world due to its advantages. Several studies have shown benefits of autodialysis in terms health relate quality of life (HRQoL) by allowing a faster return to work, better experience of the disease, patient's awareness as the first player in own healthcare and, consequently improved compliance and survival. In addition, autodialysis can provide economic benefits since it is less expensive than the treatment performed in hemodialysis centers [3, 4]. In Morocco, the practice of hemodialysis dates back to 1970, and it is common in patients who have experienced 30 years of dialysis. The prevalence of patients in renal replacement therapy (RRT) was estimated in 10,623 at the end of 2010. Over 97% of these patients were on conventional hemodialysis in nearly 180 dialysis centers [5]. Currently, there are no autodialysis centers in the country. Our study aims to assess the medical fitness of patients potentially able to achieve autodialysis, and their level of involvement in their own treatment.

## Methods

This was a descriptive, analytical multicenter study conducted from March 1^st^ to March 31^st^, 2015 in 8 of the 40 (20%) medicalized hemodialysis centers of Casablanca (Morocco) (5 private and 3 public centers). Were included patients who gave verbal consent to study participation. Ethical committee of Ibn Rochd Teaching Hospital approved this study that was conducted in two steps. The first step of the study consisted of a transversal assessment of the patients' medical potential of achieving autodialysis; after this evaluation, 556 hemodialysis patients were included. For each patient enrolled in the study, we evaluated age, general health status, autonomy, and comorbidities. Autonomy was evaluated using the Barthel index [6] by a Physical Medicine and Rehabilitation (PM & R) specialist at Ibn Rochd Teaching Hospital of Casablanca. The Barthel index includes 10 items to evaluate the patient's ability to eat, to take a shower, to dress, the anal incontinence, the bladder continence, the transferring from bed to chair, the walking, and the stairs climbing; each item was rated 10, 5, or 0 depending on the level of independence or help needs or dependence. A patient was classified as autonomous when they reached a score of 100. Comorbidities were assessed according to the observation of the diseases included in the Charlson comorbidity index [7], and those reported in the literature as the main factors of dialysis failure [8-10]. The diseases were cardiac arrhythmias, ischemic heart disease, peripheral arterial disease, and chronic obstructive pulmonary disease. A patient was considered without major comorbidities when he did not show any of these conditions. The second step of the study was the completion of a survey regarding 383 out of the 556 patients who were deemed eligible for autodialysis. They were patients under 75 years of age, in good general condition, autonomous and without significant comorbidities. The survey had to assess both the potential adherence to autodialysis by the patients, meaning if they were in favour or against autodialysis after a clear explanation by the investigator physician. Moreover, the survey was planned to evaluate the degree of the patients' involvement and awareness according to the level of patients' knowledge of the various stages of a hemodialysis session. The evaluation of the socioeconomic level of the patients was based on the criteria described in 2009 by the High Commission for Planning (HCP) [11]. Data were entered and analyzed using EPI INFO software. Quantitative variables (age, sex, mean duration of dialysis) were expressed as mean and standard deviation. The comparison of the means was performed by the student test. The categorical variables were expressed as number and percentage, and the comparison was performed by the Chi2 test or Fisher exact test. A p value <0.05 was considered significant.

## Results

The 556 patients had an average age of 54.6 ± 15 years, a female predominance (53.4%), and 66.4% of them had no occupation (Table 1). The mean duration of dialysis was 85.9 ± 78 months (Table 2). Diabetic nephropathy (22.7%) and nephroangiosclerosis (26.7%) predominated among the initial nephropathies (Table 2). The distance of round-trip between home and center was 11 km on average, with an average cost of $ 3.2. The assessment of medical capabilities for self-dialysis (Table 3) led identifying 93% of patients in good condition, 81% independent and without major comorbidities. Thus, 69% of the 556 patients evaluated was considered medically eligible for autodialysis. Patients of private centres were significantly younger, more educated and with a higher standard of living compared with the patients of public centres. Regarding the potential adherence to autodialysis, 76.5% of the 383 patients responded positively to the idea of self-dialysis, and there was no significant difference between public and private centers. Only the higher level of education was significantly associated with the potential accession to the idea of self-dialysis (Table 3).

**Table 1:** Epidemiological and hemodialysis data of patients

	Total (n=556)	Public (n=151)	Private (n=405)	p
Patients characteristics				
Age (Average±SD), year	54.6 ± 15.1	56.5±15.3	54.6±14.6	0.00
Female sex, n (%)	297 (53.4)	89 (58.9)	208 (51.4)	0.067
Socioeconomic level, n (%)				
Low	212 (38.1)	108 (71.5)	104 (25.7)	0.00
Average	319 (57.4)	43 (28.5)	276 (68.1)	
High	25 (4.5)	0 (0.0)	25 (6.2)	
Education level, n (%)				
Illiterate	196 (35.3)	65 (43.0)	131(32.3)	0.00
Primary	101(18.2)	39 (25.8)	62 (15.3)	
Secondary	143 (25.7)	38 (25.2)	105 (25.9)	
University	116 (20.9)	9 (6.0)	107 (26.4)	
Professions, n (%)				
Senior	21(3.8)	0 (0.0)	21 (5.2)	0.00
Middle-grade	73 (13.1)	4 (2.6)	69 (17.0)	
Skilled worker	33 (5.9)	4 (2.6)	29 (7.2)	
Unskilled worker	23 (4.1)	12 (7.9)	11 (2.7)	
Farmer	2 (0.4)	0 (0.0)	2 (0.5)	
Artisan/Trader	31 (5.6)	10 (6.6)	21 (5.2)	
School	4 (0.7)	1(0.7)	3 (0.7)	
Duration od dialysis (Average ± SD) month	85.9±78.1	71.7±87.2	85.9±69.9	0.00
Number of dialysis (per/week) n (%)				
3sessions/week	408 (73.4)	65 (43.1)	343 (84.7)	0.00
2sessions/week	148 (26.6)	86 (57.0)	62 (15.3)	
Initial nephropathy, n (%)				
Diabetes	126 (22.7)	18 (11.9)	108 (26.7)	0.00
Nephroangiosclerosis	138 (24.8)	17 (11.3)	121 (29.9)	
Chronic glomerulonephritis	66 (11.9)	28 (18.5)	38 (9.4)	
Tubulointerstitial	17 (3.1)	7 (4.6)	10 (2.5)	
Polycystic	33 (5.9)	7 (4.6)	26 (6.4)	
Others	31 (5.6)	3 (2.0)	28 (6.9)	
Unknown nephropathy	145 (26.1)	71 (47.0)	74 (18.3)	
Type of support, n (%)				
RAMED	201 (36.2)	141 (93.4)	60 (14.8)	0.00
NSSF	194 (34.9)	7 (4.6)	187 (46.2)	
NSPFO	85 (15.3)	1 (0.7)	84 (20.7)	
Other insurance	64 (11.5)	2 (1.3)	62 (15.3)	
Payment	12 (2.2)	0 (0.0)	12 (3.0)	
No occupation	369 (66.4)	120 (79.5)	249 (61.5)	

RAMED (Medicaid Plan) NSSF (National Social Security Fund) NSPFO (National Social Provident Fund Organizations)

**Table 2:** Data on the evaluation of medical potentials in autodialysis

	Total (n=556)	Public (n=151)	Private (n=405)	p
General health, n (%)				
Good	521 (93.7)	134 (88.7)	387 (95.6)	0.00
Bad	35 (6.3)	17 (11.3)	18 (4.4)	
Autonomy, n (%)				
Score = 100	452 (81.3)	124 (82.1)	328 (81.0)	0.801
Score <100	103(18.6)	27 (17.9)	18 (4.4)	
Comorbidity, n (%)				
Cardiac arrhythmia	35 (6.3)	10 (6.6)	25 (6.2)	0.255
Ischemic heart disease	48 (8.6)	19 (12.6)	29 (7.2)	0.043
Arteriopathy of lower limbs	13 (2.3)	7 (4.6)	6 (1.5)	0.029
COPD	11 (2.0)	2 (1.3)	9 (2.2)	0.658
Patients characteristics				
Age (Average±SD), years	50.3±13.1	50.7±15.3	48.4±14.6	0.832
Female sex, n (%)	192 (34.5)	58 (38.4)	134 (33.1)	0.783
Autodialysis adherence, n (%)	293 (52.7)	82 (54.3)	211 (52.1)	0.752

Chronic Obstructive Pulmonary Disease (COPD)

**Table 3:** Data on factors influencing adherence to autodialysis

	Adherence to autodialysis		
	Yes (n=293)	No (n=90)	p
Age (Average±SD), year	56.2±15.3	50.6±12.3	0.00
Sex, n (%)			
Male	147 (50.2)	44 (48.9)	0.463
Female	146 (49.8)	46 (51.1)	
Education level, n (%)			
Illiterate	63 (21.5)	44 (48.9)	0.00
Primary	56 (19.1)	19 (21.1)	
Secondary	98 (33.4)	13 (14.4)	
University	76 (25.9)	14 (15.6)	
Duration (Average ± SD), month	96.1±76.1	90.7±84.2	0.458
Socioeconomic level, n (%)			
Lower	110 (37.5)	34 (37.8)	0.50
Average	168 (57.3)	54 (60.0)	
High	15 (5.1)	2 (2.2)	

## Discussion

This study allowed us to reach many fundamental findings. First, the large majority (69%) of our patients with renal failure were young, especially if compared to the life expectancy of the Moroccan general population (76 years) [12], and consequently with an expected prolong duration of this chronic disease. Also, our patients were in good condition, autonomous, and without major comorbidities. These clinical features make the patients included in the current study as good candidates for a program of autodialysis treatment. A second finding is that 76.5% of the patients adhered to the idea of self-dialysis. The 66% of them was without occupation, and this condition is an additional motivation for establishing autodialysis given that it represents a treatment more economic than that performed hemodialysis centers. Indeed, cost-analysis studies reported economical advantages of self-dialysis treatments. For example, the study of Lee *et al*. showed that the practice of self-dialysis in United States reduces $20,000 a year of the cost of nursing staff compared to hemodialysis centres [3, 4]. Furthermore, self-dialysis promotes the social and professional reintegration [3, 4] and allows them to financially contributing to own healthcare. Finally, another important result of our study is that the majority of the patients are involved in achieving hemodialysis in different ways, confirming their good level of autonomy. Although this study is limited by the evaluation of the sole potential involvement of the RRT patients, its results contribute to identifying the patients' clinical profile most suitable for a program of autodialysis in Morocco. While ensuring long-term clinical outcomes, this program aims not only at reducing the expenditure of the whole healthcare system but may lead to improved outcomes.

## Conclusion

The practice of autodialysis represents a constructive way to involve RRT patients in their own treatment, increase the HRQoL, and reduce the costs. This study highlighted that the patients' profile makes them medically eligible for this treatment strategy. Therefore, the implementation of an autodialysis program in the Morocco territory is advisable for the healthcare expenditure control maintaining clinical effectiveness of the RRT treatment.

### What is known about this topic

- The world practice of autodialysis dates back several decades particulary in developed 197 countries. This kind of dialysis is rarely practiced in developing countries such as 198 Morocco.

### What this study adds

- This study prove that autodialysis set up is possible in Morocco.

## Competing interests

The authors declare no competing interests.
